# Model and Energy Bounds for a Two-Dimensional System of Electrons Localized in Concentric Rings

**DOI:** 10.3390/nano14201615

**Published:** 2024-10-10

**Authors:** Orion Ciftja, Josep Batle, Mahmoud Abdel-Aty, Mohammad Ahmed Hafez, Shawkat Alkhazaleh

**Affiliations:** 1 Department of Physics, Prairie View A&M University, Prairie View, TX 77446, USA; 2 Departament de Física and Institut d’Aplicacions Computacionals de Codi Comunitari (IAC3), University of the Balearic Islands, E-07122 Palma de Mallorca, Spain; jbv276@uib.es; 3 CRISP—Centre de Recerca Independent de sa Pobla, E-07420 Mallorca, Spain; 4 Mathematics Department, Faculty of Science, Sohag University, Sohag 82524, Egypt; mabdelaty@zewailcity.edu.eg; 5 Deanship of Graduate Studies and Research, Ahlia University, Manama P.O. Box 10878, Bahrain; 6 Department of Civil Engineering, Faculty of Engineering, INTI International University, Nilai 71800, Malaysia; 7 Department of Mathematics, Faculty of Science and Information Technology, Jadara University, Irbid P.O. Box 733, Jordan

**Keywords:** nanoscience, nanotechnology, quantum systems, concentric rings, semi-classical approximation, energy bounds

## Abstract

We study a two-dimensional system of interacting electrons confined in equidistant planar circular rings. The electrons are considered spinless and each of them is localized in one ring. While confined to such ring orbits, each electron interacts with the remaining ones by means of a standard Coulomb interaction potential. The classical version of this two-dimensional quantum model can be viewed as representing a system of electrons orbiting planar equidistant concentric rings where the kinetic energy may be discarded when one is searching for the lowest possible energy. Within this framework, the lowest possible energy of the system is the one that minimizes the total Coulomb interaction energy. This is the equilibrium energy that is numerically determined with high accuracy by using the simulated annealing method. This process allows us to obtain both the equilibrium energy and position configuration for different system sizes. The adopted semi-classical approach allows us to provide reliable approximations for the quantum ground state energy of the corresponding quantum system. The model considered in this work represents an interesting problem for studies of low-dimensional systems, with echoes that resonate with developments in nanoscience and nanomaterials.

## 1. Introduction

Nanoscience is the science of very small objects and materials that have sizes commensurate with those of atoms and molecules. It is one of the current topics of great interest in all of science, touching on physics, biology, chemistry as well as materials science and engineering. By advancing our understanding of materials at this scale, scientists are gaining a better understanding of how various properties emerge. This knowledge can be used to design better structures with great potential use. Research on nanostructures and nanomaterials has given rise to the development of novel technologies and has enhanced the progress of nanoscience towards unforeseen scientific frontiers. For instance, this progress is clearly seen in the world of microelectronics, where smaller has always meant more components per chip, a faster response, lower cost, lower power consumption, and improved device performance. This trend towards miniaturization and the production of microelectronic chips with dimensions in the nanometer range seems to continue unabated [1,2,3,4,5,6,7].

Within the framework of various nanostructures, the family of two-dimensional (2D) nanomaterials stands out because of the unique properties associated with their lower dimensionality. Typically, such materials are composed of thin layers that may have a thickness of one atomic layer. Differently from their bulk counterparts, these materials have a lot of atoms on their surface. These atoms behave differently from the atoms in bulk. As a result, a 2D material may behave quite differently from its bulk counterpart. One of the must famous examples of this category is graphene. As one of the most important 2D materials, graphene has unique properties that have led to its widespread use in various industries. In particular, the behavior of electrons in 2D graphene samples, as well as the properties of electrons in other low-dimensional structures such as 2D semiconductor systems, has been the object of intensive research for the past few decades. The competition between confinement, spin effects, delocalization, and strong Coulomb repulsion between electrons provides a fertile ground for observation of interesting physical phenomena pertaining to 2D systems of electrons under various conditions [8,9,10,11,12,13,14,15,16,17,18,19,20,21].

It is worth noting that the study of electron correlations in 2D systems has always been one of the main goals of quantum mechanics and condensed matter physics. Multi-pronged efforts in this direction run in parallel with new theories and models in this fast-evolving research area [22,23]. Recently, fabrication of GaAs/AlGaAs double concentric quantum rings [24,25] has enabled, for instance, the study of the Aharonov–Bohm effect [26] as well as the role of Coulomb interaction in magnetic systems [27]. The introduction of minimal models for these intricate quantum systems is a very desirable feature given the complexity of the phenomena that are observed in these structures. Quasi-exact quantum approaches have been successfully applied to different geometries of concentric [28] and parallel [29] quantum rings. However, quantum treatment becomes very difficult when one decides to carry out ab initio calculations and the number of particles is not small. For a situation like this, one may resort to a classical description that may provide very useful insights with less difficulty. For instance, classical studies of 2D systems of interacting electrons confined by a harmonic potential as well as electrons confined to a single circle [30,31,32,33] have shed light on their peculiar properties. For instance, the Thomson problem [34] has been extensively studied from very different perspectives in the literature [35,36,37,38].

In this work, we introduce a model of interacting electrons confined to a 2D planar system. The system consists of an arbitrary number of interacting electrons localized in equidistant circular rings. The electrons are considered spinless and each of them occupies one orbit. The novelty of this model is the fact that the orbits of the electrons are predefined. As a result, no external confinement is required. The ensuing model for the electrons, somehow, mimics that of a 2D classical atom, but without the presence of an attractive central force. The motivation for studying such a model from both a quantum and a classical perspective is two-fold: (i) we provide a brand new (to our knowledge) discrete problem to solve; and (ii) we shed light on the properties of an untractable quantum problem by using classical tools. As we show in this work, the corresponding equilibrium solutions of the 2D classical model under consideration are closely related to the counterpart quantum problem of searching for the ground state energy.

The present contribution is divided as follows: In Section 2, we introduce the classical model to study. In Section 3, we define lower and upper bounds to the ground state energy of the quantum model. The main results for the 2D classical model counterpart as well as related quantum energy bounds are shown in Section 4. An extension of the classical Coulomb model to include Hooke’s law interaction is discussed in Section 5. Finally, some brief conclusions are drawn in Section 6.

## 2. Classical Model

**Figure 1:**
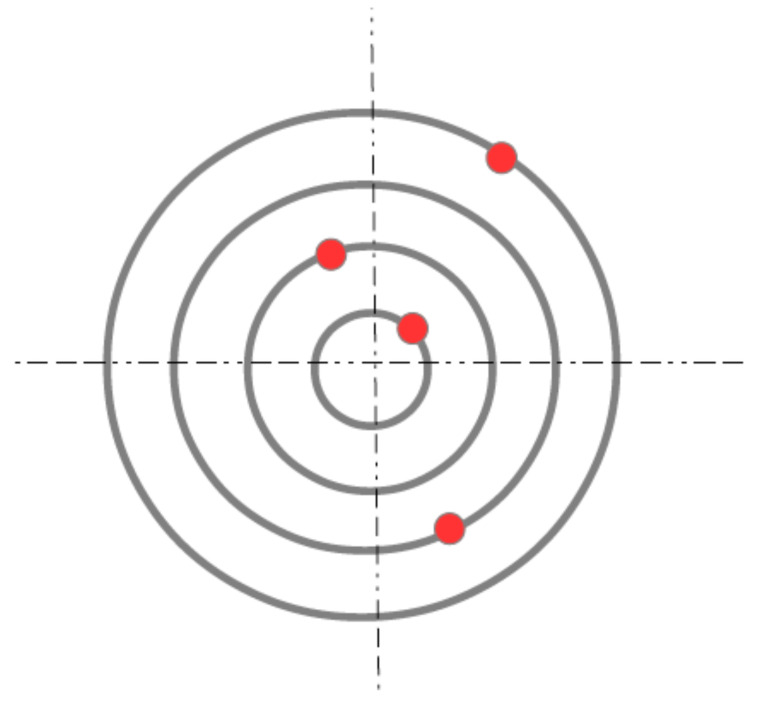
(Color online) Relative position of the rings, each one containing one electron. See text for details.

We consider a system of N≥2 electrons with charge, −e(e>0) confined to circular, concentric orbits that are equally spaced. In other words, the first electron is forced to move in a circular ring with radius $R_{1}=R$; the second electron moves in a circular orbit with radius $R_{2}=2R$; and, similarly, the *N*-th electron is confined to a ring with radius $R_{N}=NR$. A schematic view of the model is shown in Figure 1.

The total Coulomb interaction energy of the system is written as

(1) $$U_{N}\;=\;\sum_{1\leq i<j\leq N}\frac{k\;e^{2}}{\sqrt{R_{i}^{2}+R_{j}^{2}-2\;R_{i}\;R_{j}\cos\left(\phi_{i}-\phi_{j}\right)}}\;,$$

where $R_{i}$ is the radial coordinate of the *i*-th electron, $\phi_{i}$ is its azimuthal (polar) angle, and *k* is Coulomb’s electric constant. The equilibrium of this purely electrostatic system is reached when the total energy reaches a global minimum. Taking into account the fact that orbits have fixed radii ($R_{i}=i\;R$; i=1,2,…N), we write a more compact dimensionless object to optimize:

(2) $${\tilde{U}}_{N}\equiv \frac{U_{N}}{\left(\frac{k\;e^{2}}{R}\right)}\;=\;\sum_{1\leq i<j\leq N}\frac{1}{\sqrt{i^{2}+j^{2}-2\;i\;j\;\cos\left(\phi_{i}-\phi_{j}\right)}}.$$

For simplicity and for direct comparison of the results of this classical model to the quantum case counterpart, we assume that the radius of the first ring (namely, parameter *R*) is $R=a_{B}$, where $a_{B}$ is the Bohr radius. This means that the chosen unit of energy is the atomic unit, $k\;e^{2}/a_{B}$ (a Hartree), which is a commonly used unit in quantum atomic physics. As matter of fact, atomic units (with $a_{B}$ as unit of length and $k\;e^{2}/a_{B}$ as unit of energy) are used for the quantum counterpart case. The complexity of having to deal with the expression in Equation (2) lies in the fact that the electrons are not positioned in the same ring. Nevertheless, there are certain bounds that we can obtain by looking at the functional form of Equation (2) even before embarking on the process of minimizing the total energy function. First, we notice that

(3) $$\sum_{i=1}^{N-1}\sum_{j=i+1}^{N}\frac{1}{j+i}\;\leq \;\;{\tilde{U}}_{N}\;\;\leq \sum_{i=1}^{N-1}\sum_{j=i+1}^{N}\frac{1}{j-i}\;.$$

For the case of two electrons, we have $\frac{1}{3}\leq {\tilde{U}}_{2}\leq 1$. The equilibrium solution in this case is trivial and coincides with the lower energy bound. The case of three particles reads as $\frac{1}{3}+\frac{1}{4}+\frac{1}{5}\leq {\tilde{U}}_{3}\leq 1+\frac{1}{2}+1$. This result suggests that the optimal solution of Equation (2) should be found within the [0.783,2.5] range. The actual minimum energy value that we found numerically (0.8805) is quite close to the lower energy bound (0.783). Thus, we reasonably expect the latter to be of major relevance as we proceed further with increasing the values of *N*. The lower energy bound does not seem to correspond to any easily identifiable physical configuration. On the other hand, the upper energy bound represents the maximum possible value of the quantity in Equation (2). This value corresponds to having all electrons aligned in the same direction, similar to the case of one-dimensional (1D) ionic crystals [39,40].

In addition to such observations for small values of *N*, we also point out that the energy bounds are related to the harmonic series, $H_{M}$, truncated at some value *M*. Therefore, let us derive explicit bounds for the optimal energies of Equation (2) via Equation (3) and study the corresponding asymptotic behavior for a large *N*. It turns out that the lower energy bound can be written in terms of the digamma function, ψ(N)=H(N−1)−γ, with H(N−1) being the summation of the harmonic series up to N−1 terms and γ the Euler constant. After some algebra, the lower energy bound in Equation (3) is obtained *exactly*. The upper energy bound is found to be exact as well. The final result reads

(4) $$\begin{array}{c} NH\left(2N-1\right)-\left(N+1\right)H\left(N+1\right)+\frac{1}{4}H\left(N+\frac{1}{2}\right)\\ +\frac{11}{60}+\frac{\ln2}{2}\leq \;\;{\tilde{U}}_{N}\;\;\leq 1+N\left[H\left(N-1\right)-1\right]\;. \end{array}$$

At this juncture, let us divide by *N* all terms in the above inequality, taking into account that H(N)=lnN+γ+O(1/N) and that N≫1. Under these conditions, we have

(5) $$\ln2\;+\;\frac{\ln N}{4N}<\;\;\frac{{\tilde{U}}_{N}}{N}\;\;<\ln N\;.$$

This means that the energy per particle will be no greater than a logarithmic function, lnN, whatever is the minimum of the classical energy. This is a common result for planar structures in electrostatics studies [41,42]. The significance of Equation (5) lies in the fact that it states the existence of a minimum (and a maximum) for the classical approach to the problem. However, the lower bound is not tight enough. If it was tight enough one might have argued that full optimization of the energy could be avoided, at least as a first crude step. However, as we found out, one must minimize the total energy numerically, in our case by means of a simulated annealing procedure, in order to obtain meaningful accurate results. Therefore, the minimum classical energy for systems with arbitrary values of *N* is obtained numerically and is denoted as $E_{C}^{min}=min\left\{{\tilde{U}}_{N}\right\}$. The results obtained for both the classical and quantum model counterparts are discussed in more detail in Section 4.

## 3. Quantum Model—Ground State Energy

Let us consider the quantum counterpart to the model introduced earlier. The problem setup assumes that the interacting electrons confined in equidistant 2D concentric rings are spinless (fully spin polarized). This is a simplification because the electrons possess two possible spin states. In the absence of interactions, it is evident that the wave function of each electron is confined in its respective ring, and thus, there is no overlap. In reality, the effect of the spin can be negligible in situations that are more realistic than our model as long as the inter-ring distance is large enough to lead to only a small overlap between the wave functions of respective electrons in neighboring rings. The exchange effect, and the effect of tunneling between the rings, can be neglected even in realistic systems when this condition is satisfied. The idea is to prove that there is an energy bound for the quantum case, which is also a bound for the minimum energy of the classical configuration. To this effect, let us look at the quantum mechanical problem for N=2 electrons situated at circular rings with radii $R_{1}$ and $R_{2}$, respectively. The quantum Hamiltonian is written as

(6) $$\hat{H}\left({\vec{R}}_{1},{\vec{R}}_{2}\right)=-\frac{\hbar^{2}}{2\;m_{e}}\left(\nabla_{1}^{2}+\nabla_{2}^{2}\right)+\frac{k\;e^{2}}{|{\vec{R}}_{1}-{\vec{R}}_{2}|}\;,$$

where ${\vec{R}}_{i}=\left(x_{i},y_{i}\right)$ is the 2D vector position of the *i*-th electron in the *i*-th ring, $\nabla_{i}^{2}$ is the 2D Laplacian operator, $m_{e}$ is an electron’s mass, and *ℏ* is the reduced Planck’s constant.

As stated earlier, we use atomic units in this work. The stationary Schrödinger equation in atomic units reads

(7) $$\begin{array}{c} -\frac{1}{2R_{1}^{2}}\frac{\partial^{2}}{\partial \phi_{1}^{2}}\Psi \left(\phi_{1},\phi_{2}\right)-\frac{1}{2R_{2}^{2}}\frac{\partial^{2}}{\partial \phi_{2}^{2}}\Psi \left(\phi_{1},\phi_{2}\right)\\ +\frac{1}{d(\phi_{1},\phi_{2})}\Psi \left(\phi_{1},\phi_{2}\right)\;=\;E\;\Psi \left(\phi_{1},\phi_{2}\right)\;, \end{array}$$

where $\phi_{1,2}$ are the azimuthal (polar) angles and $d\left(\phi_{1},\phi_{2}\right)=\left|{\vec{R}}_{1}-{\vec{R}}_{2}\right|=\sqrt{R_{1}^{2}+R_{2}^{2}-2\;R_{1}\;R_{2}\;\cos\left(\phi_{1}-\phi_{2}\right)}$ is the separation distance between the pair of electrons localized in two different rings, $R_{1}$ and $R_{2}$. One way to solve Equation (7) by preserving the periodicity is to span the unknown solution $\Psi (\phi_{1},\phi_{2})$ in the basis functions of two non-interacting particles, one in each ring, and then, truncate the expansion to N+1 terms (*N* even):

(8) $$\Psi \left(\phi_{1},\phi_{2}\right)\;=\;\sum_{m=-\frac{N}{2}}^{\frac{N}{2}}\sum_{n=-\frac{N}{2}}^{\frac{N}{2}}c_{m,n}\;\frac{e^{i\;m\;\phi_{1}}e^{i\;n\;\phi_{2}}}{2\pi \sqrt{R_{1}R_{2}}}\;.$$

By substituting Equation (8) into Equation (7), multiplying both sides by $\frac{1}{2\pi \sqrt{R_{1}R_{2}}}e^{-ik\phi_{1}}e^{-il\phi_{2}}$, and integrating over $\left\{\phi_{1},\phi_{2}\right\}$ one obtains

(9) $$\begin{array}{cc} \sum_{k=-\frac{N}{2}}^{\frac{N}{2}}\sum_{l=-\frac{N}{2}}^{\frac{N}{2}} & [\left(\frac{m^{2}}{2R_{1}^{2}}+\frac{n^{2}}{2R_{2}^{2}}\right)\delta_{k,m}\delta_{l,n}+\langle kl|\frac{1}{d}|mn\rangle \\ & -E\;\delta_{k,m}\delta_{l,n}]\;c_{k,l}\;=\;0\;, \end{array}$$

for $m,n\;=\;-\frac{N}{2},..,\frac{N}{2}$. Let us denote as $H_{klmn}$ the term in the first line in Equation (9). Solving Equation (9) for $c_{k,l}$ is tantamount to providing an approximate solution to Equation (7) for the ground or excited states, with an accuracy that increases as the number of terms in the basis set increases. The matrix element for the Coulomb interaction term in Equation (9) reads explicitly as

(10) $$\langle kl|\frac{1}{d}|mn\rangle \;=\;\frac{1}{4\pi^{2}}\int_{0}^{2\pi }\int_{0}^{2\pi }d\phi_{1}d\phi_{2}\;\frac{e^{i\left(m-k\right)\phi_{1}}e^{i\left(n-l\right)\phi_{2}}}{d(\phi_{1},\phi_{2})}\;.$$

The set of Equation (9) for $c_{k,l}$ does not read yet as a standard eigenvalue–eigenstate problem. For this, we must transform $H_{klmn}⟶A_{ij}$ and $c_{k,l}⟶g_{j}$, with $i,j\;=\;1,\ldots ,{\left(N+1\right)}^{2}$ using $i=\left(m+\frac{N}{2}\right)\left(N+1\right)+\left(n+\frac{N}{2}\right)+1$ and $j=\left(k+\frac{N}{2}\right)\left(N+1\right)+\left(l+\frac{N}{2}\right)+1$∀(k,l,m,n). With this transformation, we have the usual eigenvalue–eigenvector problem:

(11) $$\sum_{j=1}^{{\left(N+1\right)}^{2}}\left(A_{ij}-E\;\delta_{ij}\right)\;g_{j}\;=\;0,$$

where $i\;=\;1,2,\ldots ,{\left(N+1\right)}^{2}$. The eigenvalues give the energy spectrum of the system. In order to find the eigenvectors, the inverse transformation $g_{j}⟶c_{k,l}$ can be proved to be unique. In other words, given *j* and *N*, we can find a sole couple (k,l).

Now, since we are looking for a bound to the quantum ground state energy, the kinetic energy terms in Equation (9) can be dropped (since they are always non-negative). The Coulomb energy term is bounded by virtue of the Cauchy–Schwarz inequality for integrals:

(12) $$\langle kl|\frac{1}{d}|mn\rangle \;\leq \;E_{QM}^{bound}\;\equiv \;\frac{1}{4\pi^{2}}\int_{0}^{2\pi }\int_{0}^{2\pi }d\phi_{1}d\phi_{2}\;\frac{1}{d(\phi_{1},\phi_{2})}.$$

Thus, the eigenvalue problem in Equation (9) gives rise to a total energy which is larger than the exact potential energy, which we denote as $E_{QM}^{0}$. This is easily understood by recalling that the total energy includes non-negative kinetic energy terms. In other words, for the general *N*-particle problem, we can write that

(13) $$E_{QM}^{0}\;<\;E_{QM}^{bound}=\sum_{1\leq i<j\leq N}\frac{1}{4\pi^{2}}\int_{0}^{2\pi }\int_{0}^{2\pi }d\phi_{i}d\phi_{j}\;\frac{1}{d(\phi_{i},\phi_{j})}\;.$$

The classical equilibrium energy for *N* electrons in concentric rings, $E_{C}^{min}$, is also bounded by $E_{QM}^{bound}$, because the latter is nothing more than the average over all inverses of the inter-particle distances. Since the minimum classical energy configuration is always less than the average, we have $E_{C}^{\min}\;<\;E_{QM}^{bound}$. For the exactly tractable case of two electrons ($R_{1}=a_{B}$ and $R_{2}=2\;a_{B}$), we have $E_{C}^{\min}=\frac{1}{3}=0.333$ and $E_{QM}^{bound}=0.536$, while $E_{QM}^{0}=0.483$. The exact proof that the classical minimum energy is smaller than its exact quantum counterpart ($E_{C}^{\min}\;<\;E_{QM}^{0}$) is a little bit more involved, but it has been extensively checked numerically. The following lemma gives a sense of how the proof is obtained for the case of a one-particle quantum problem.

**Lemma** **1.** *The classical (lowest) equilibrium energy is always a lower bound for the corresponding quantum energy of a system. To this effect, let us assume that we have a quantum system described by the quantum Hamiltonian (written in atomic units):*

(14) $$\hat{H}=-\frac{1}{2}\Delta +V\left(x\right)\;,$$

*where* Δ *is a d-dimensional Laplace operator on* $R^{d}$ *space and* V(x) *is a real-valued potential with* $x\in R^{d}$*. Assume that* V(x) *is bounded below and that a minimum exists:*

(15) $$V_{min}=\min_{x\in R^{d}}V\left(x\right)\;.$$

**Proof.**  For all normalized wave functions, ψ, one has

(16) $$\langle \psi ,\hat{H}\psi \rangle \geq V_{min}\;.$$

To be more technical and consistent with the quantum rules, we must say that the wave function, ψ, should be in the domain of $\hat{H}$ or the quadratic form domain of $\hat{H}$ (where the energy of the system is finite), and the operator $\hat{H}$ should be self-adjoint. Here, $\langle f,g\rangle =\int \bar{f(x)}g\left(x\right)\;dx$ is the usual scalar product on the Hilbert space $L^{2}\left(R^{d}\right)$, where the overline symbol means complex conjugation.

Having reached this stage, we have

(17) $$\begin{array}{ccc} \langle \psi ,\hat{H}\psi \rangle & = & \langle \psi ,-\frac{1}{2}\Delta \psi \rangle \\ & & +\langle \psi ,V\psi \rangle =\frac{1}{2}\int {\left|\nabla \psi \left(x\right)\right|}^{2}\;dx\\ & & +{\int V\left(x\right)|\psi \left(x\right)|}^{2}\;dx\\ & & \geq {\int V\left(x\right)|\psi \left(x\right)|}^{2}\;dx\;. \end{array}$$

To obtain the above results, we used the fact that the kinetic energy term is positive. At this juncture, we point out that ${\left|\psi \left(x\right)\right|}^{2}$ is non-negative. Therefore, for all $x\in R^{d}$ we have the following lower bound:

(18) $${V\left(x\right)|\psi \left(x\right)|}^{2}\geq V_{min}{\left|\psi \left(x\right)\right|}^{2}\;,$$

which can be easily checked by recalling that $V\left(x\right)\geq V_{min}$. By using the fact that $V_{min}$ is a constant, we have

(19) $$\begin{array}{ccc} {\int V\left(x\right)|\psi \left(x\right)|}^{2}\;dx & \geq & \int V_{min}{\left|\psi \left(x\right)\right|}^{2}\;dx\\ & & =V_{min}\int {\left|\psi \left(x\right)\right|}^{2}\;dx=V_{min}\;, \end{array}$$

since we are assuming that ψ is properly normalized. So, for any normalized quantum wave function we have

(20) $$\langle \psi ,\hat{H}\psi \rangle \geq V_{min}\;.$$

The Rayleigh–Ritz principle now says that the ground state energy of the spectrum is given by

(21) $$E_{0}=\min_{\psi \in L^{2}\left(R^{d}\right),{∥\psi ∥}^{2}=1}\langle \psi ,\hat{H}\psi \rangle \geq V_{min}\;,$$

which concludes the proof. □

One can make sense of the main conclusion of this lemma by intuitively appealing to the role played by Heisenberg’s uncertainty principle for such a problem.

## 4. Results

### 4.1. Bounds to Classical Energy

Finding the minimum value, $E_{C}^{\min}$, of the quantity in Equation (2) is a very difficult global minimization problem. However, this goal may be achieved numerically by using tools originating from molecular dynamics simulations. In our case, we employ the well-known simulated annealing approach [43]. This robust numerical approach contains a mechanism that allows for numerical searches to eventually escape local minima and thus, enables one to identify the global energy minimum in a reliable manner.

**Figure 2:**
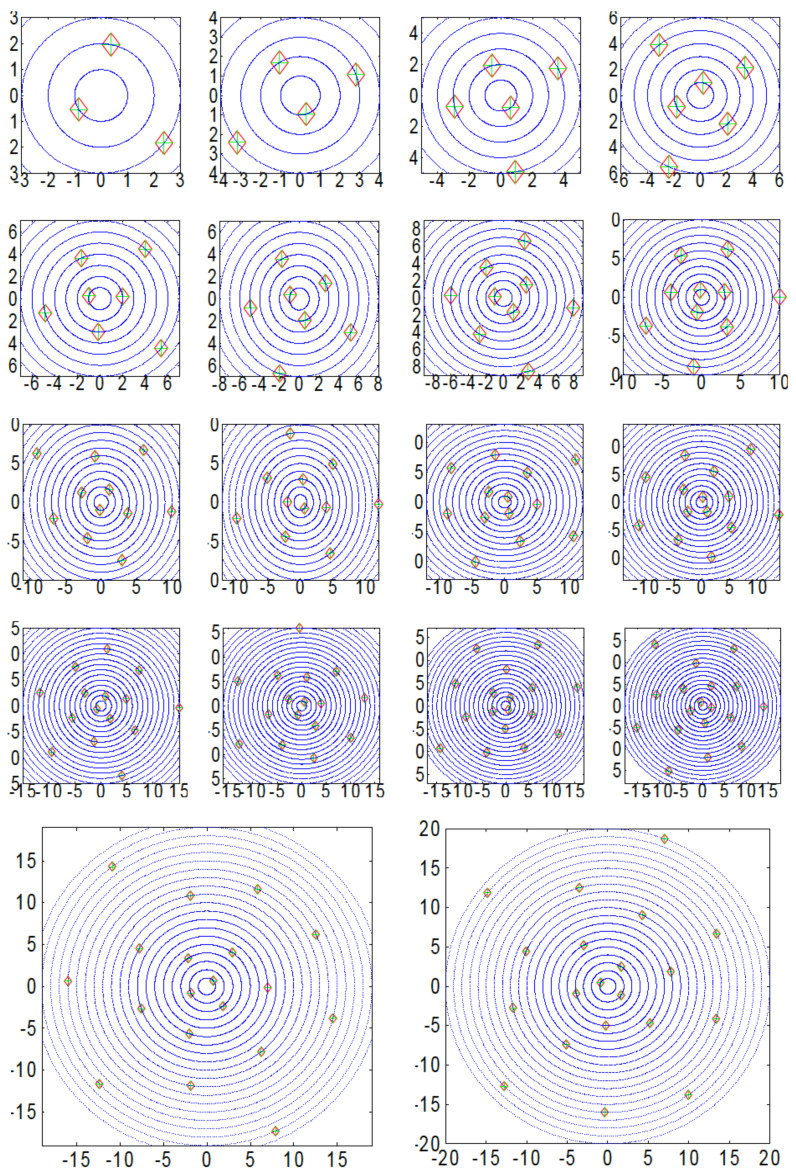
(Color online) Equilibrium configuration for systems with N=3−20 electrons. Distances are given in atomic units, where it is assumed that $R=a_{B}$. See text for details.

Results for the minimum total classical energies and configurations for systems with N=3−20 electrons are shown in Table 1 and Figure 2, respectively. One notices that the electrons seem to spread homogeneously in the plane, but, while doing so, they preserve some parallel alignments to the diameters of the concentric circumferences.

**Figure 3:**
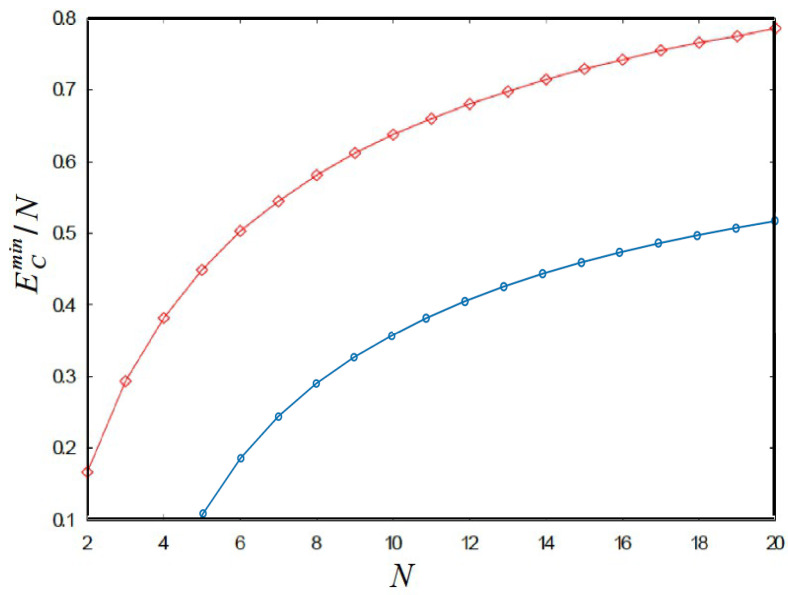
(Color online) Minimum classical energy per particle $E_{C}^{\min}/N$ (upper curve) as a function of *N* for systems ranging from N=2 to N=20 electrons. Its lower bound from Equation (5) is plotted as the lower curve for comparison. Note that the lower curve steadily tends to ln2 as *N* increases. The energy is given in atomic units of $k\;e^{2}/a_{B}$, where $R=a_{B}$ is assumed. See text for details.

Figure 3 depicts the evolution of the classical energy per particle, $E_{C}^{\min}/N$, as a function of *N* for N=2−20 electrons.

It is worth mentioning that we observed that the minimum energy per particle, $E_{C}^{\min}/N$, obtained from the annealing simulations is closely followed from below by the energy bound, $\ln2+\frac{\ln N}{4N}$, whereas the upper bound is not tight. Thus, it seems that the behavior of the total energy, $E_{C}^{\min}$ is to be found between $N\ln2+\frac{\ln N}{4}$ and NlnN. A stability analysis performed via $\Delta \mu =E_{N+1}+E_{N-1}-2\;E_{N}$ does not reveal any special packing or magic number, at least for the system sizes currently considered. We also remark that finding the global energy minimum for this model turned out to be a very challenging simulation problem because of the peculiar constraints imposed in the model. This explains our computational limitations of not being able to go to very high values of *N* in our simulations.

### 4.2. Bounds to Quantum Energy

**Figure 4:**
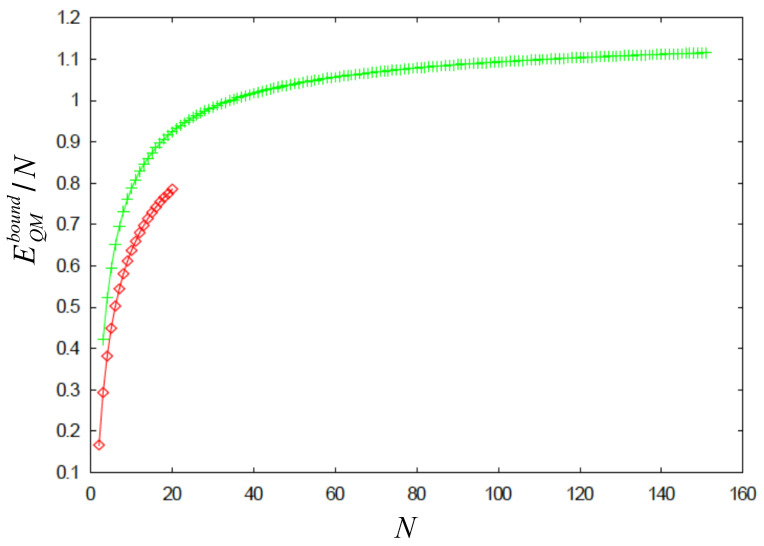
(Color online) Energy per particle, $E_{QM}^{bound}/N$, as a function of *N* (upper curve). The classical counterpart $E_{C}^{\min}/N$ is also shown for comparison (lower curve). The energy is given in atomic units of $k\;e^{2}/a_{B}$, where $R=a_{B}$ is assumed. See text for details.

In view of the previous results that we found, it is reasonable to expect that the quantum ground state of *N* electrons, each in its respective fixed ring, may be well described by a many-electron wave function that has peaks around the equilibrium positions of the classical counterpart system. In order to find the quantum energy bound, $E_{QM}^{bound}$, defined in Equation (13), we calculate the mean value of the inverse distance, $\frac{1}{d(\phi_{i},\phi_{j})}$, for all the N(N−1)/2 possible pairs of electrons. The resulting quantum energies per particle, $E_{QM}^{bound}/N$, are depicted in Figure 4 as a function of *N* (upper curve).

The classical equilibrium positions are obtained by the simulated annealing approach. While it is possible to find the global energy minimum for systems with N>20 electrons, we found that this process comes at a huge computational cost. The constraints imposed in our model make the calculation of the global energy minimum difficult even for the case of N=3 electrons (the case of N=2 electrons is the only one where we can identify an analytical result). Since we wanted to display each of the individual equilibrium positions, a sort of Mendeleev table, we stopped at N=20, since the tendency is more or less clear. In this way we also avoided the pitfalls of additional costly simulations in which we would have been limited in accuracy by our computational power.

The bound energy curve for the quantum scenario does not correspond to a logarithm function. It is similar to the bound for classic equilibrium energies, namely, $\ln2+\frac{\ln N}{4N}$, plus some additive terms. This fact would imply that, for large *N* the chemical potential for either the classical or the quantum system would be independent of *N*. In any case, an important result of the present work is the observation that the quantum energy of the system lies between two given precise bounds. This means that we have encountered a quantum system that allows the identification of distinct upper and lower bounds to the ground state energy.

## 5. Moshinsky-like Systems with Coulomb Interaction

The generalized Moshinsky system (or atom) [44] consists of *N* harmonically interacting particles confined by an overall external isotropic harmonic potential. Let us now consider a modification of the previous 2D planar ring model. The modification concerns the addition of a harmonic oscillator coupling between all electrons, $\frac{m_{e}}{2}\omega^{2}{\left({\vec{R}}_{i}-{\vec{R}}_{j}\right)}^{2}$, where $m_{e}$ is the mass of an electron and ω is the angular frequency. This harmonic oscillator coupling is in addition to the already-present Coulomb interaction term. No external confinement is added since the electrons are kept confined in fixed rings in both the original or presently modified model. A quantum treatment of this modified problem is only partially possible for two electrons [44]. Therefore, let us deal here with lower energy bounds to the quantum case by employing classical tools. For such a situation, the total energy of the system would be written as

(22) $$U_{N}\;=\;\sum_{1\leq i<j\leq N}\left(\frac{k\;e^{2}}{d_{ij}}\;+\;\frac{m_{e}}{2}\;\omega^{2}\;d_{ij}^{2}\right)\;,$$

where $d_{ij}=R\sqrt{i^{2}+j^{2}-2\;i\;j\cos\left(\phi_{i}-\phi_{j}\right)}$ is the usual pair separation distance between pairs of electrons. Let us write the total energy of the system in atomic units:

(23) $${\tilde{U}}_{N}\;=\;\sum_{1\leq i<j\leq N}\left(\frac{1}{d_{ij}}\;+\;\frac{1}{2}\;\Omega^{2}\;d_{ij}^{2}\right)\;,$$

where Ω is some rescaled dimensionless angular frequency. The rest is under the same conditions as before, namely, it is assumed that we have *N* electrons confined to circular, concentric rings that are equally spaced ($R_{1}=R,R_{2}=2\;R,..,R_{N}=N\;R$), with only one electron per ring, and we take $R=a_{B}$, where $a_{B}$ is the Bohr radius. Equilibrium is reached when the quantity in Equation (23) has a global minimum.

There are two opposite regimes to consider depending on the strength of Ω. When Ω is small enough, a repulsive regime dominates. When Ω is big enough, all electrons seem to align along the same direction, having a total *exact* energy:

(24) $$E_{N}^{\Omega }\;=\;\frac{1}{2}\;\Omega^{2}\;R^{2}\;\frac{N^{2}\left(N^{2}-1\right)}{12}\;+\;\frac{1}{R}\left(1+N[H(N-1)-1]\right)\;.$$

**Figure 5:**
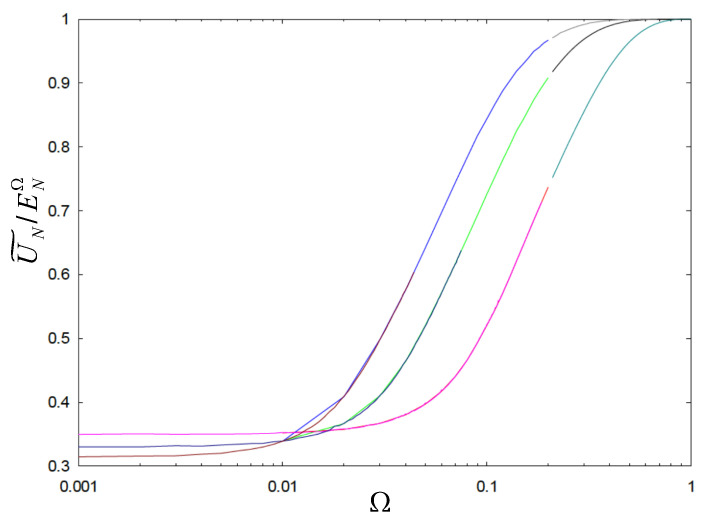
(Color online) Equilibrium energies as ${\tilde{U}}_{N}/E_{N}^{\Omega }$ versus Ω for N=5 (upper curve), N=10 (middle curve), and N=15 (lower curve). The change in regime occurs quite abruptly. See text for details.

In order to gauge the behavior of the minimum equilibrium energy as a function of frequency, Ω, we performed numerical simulations using the simulated annealing approach to find the minimum energy corresponding to the expression in Equation (23). We express this quantity as a ratio to $E_{N}^{\Omega }$ and study its dependence as a function of Ω. For such a choice, a value close to one indicates that the full harmonic oscillator regime has become dominant over the repulsive Coulomb term. The results of the calculations are depicted in Figure 5 for N=5,10, and 15 electrons.

Note that as Ω→0, the energies tend to the electrostatic equilibrium values. Also, notice how sudden the change is. Thus, for the case of this Moshinsky-like 2D classical system, the concomitant equilibrium values are very sensitive to the oscillator frequency. The question of what is any upper energy bound for the total or potential energy in the quantum setting of this case is quite intricate. The argument using the Cauchy–Schwarz inequality may not hold in this scenario. In the classical case, it looks like Equation (24) represents an upper bound (when all particles are aligned along the same direction). However, in the quantum setting, we do not think that there is such an upper limit that can be easily identified.

## 6. Conclusions

To summarize, in this work we consider a 2D system of interacting electrons confined in equidistant planar circular rings. The electrons are considered spinless. A constraint is set pertaining to the localization of electrons in specific rings by mandating that one ring must be occupied by a single electron. While being confined to such rings, the electrons interact with each other via the standard Coulomb interaction potential. Both the quantum and the classical version of this model are considered. The intricacies of quantum mechanics and the presence of electron correlations does not permit analytic results. Therefore, a key idea of this work is to extract valuable information for the system by looking at energy bounds from both a quantum and a classical perspective. For example, in a quantum scenario, one may discard the quantum kinetic energy term when looking for lower energy bounds. At this juncture, it is worth discussing possible realistic settings where the present model, both the pure repulsive Coulomb interaction and the repulsive–attractive situation (with the added harmonic confinement that induces localization), can be useful. The discussed ring model is definitely useful for modeling a nanowire or a line dislocation since it can be made periodic solely in 1D. Furthermore, a collection of rings could be related to an array of 1D crystals. It is known that dealing with a classical counterpart to a 2D quantum model can be extremely useful [45]. In the present case, this approach leads to a global minimization problem for the energy of the system that can be handled numerically by using the simulated annealing method. By using numerical simulations we were able to accurately obtain the equilibrium position configuration and energy of the system. Then, we compared such classical energy bounds to the quantum case counterpart. The analogy between the classical discrete problem and its quantum counterpart is discussed from the perspective of energy bounds. Along these lines, we obtained analytic expressions for the energy bounds for both classical and quantum models. Furthermore, we also studied the concomitant behavior of the system as a function of the number of electrons. We believe that the correspondence found between equilibrium energies in classical systems and quantum energy bounds may open the door to studying complex quantum many-body systems by means of simpler tools in a way that leads to novel ideas. 

## Figures and Tables

**Table 1:** Minimum total classical energy, $E_{C}^{min}$, for a given number of electrons. The energy is given in atomic units of $k\;e^{2}/a_{B}$, where $R=a_{B}$ is assumed. See text for details.

*N*	$E_{C}^{\min}$	*N*	$E_{C}^{\min}$
3	0.8805	12	8.16254
4	1.5266	13	9.06952
5	2.2442	14	10.0028
6	3.0176	15	10.9343
7	3.8100	16	11.8688
8	4.6470	17	12.8314
9	5.5058	18	13.7806
10	6.3744	19	14.7164
11	7.2543	20	15.7123

## Data Availability

The data presented in this study are available upon request from the authors.
